# Titanium Dioxide Nanoparticles are not Cytotoxic or Clastogenic in Human Skin Cells

**DOI:** 10.4172/2161-0525.1000239

**Published:** 2014-09-06

**Authors:** Cynthia L Browning, Therry The, Michael D Mason, John Pierce Wise

**Affiliations:** 1 Wise Laboratory of Environmental and Genetic Toxicology, University of Southern Maine, Portland ME 04103, USA; 2 Maine Center for Toxicology and Environmental Health, University of Southern Maine, Portland ME 04103, USA; 3 Graduate School of Biomedical Science and Engineering, University of Maine, Orono ME 04469, USA

**Keywords:** Titanium dioxide, Nanotoxicology, Clastogenicity, Chromosome aberration, Skin fibroblasts

## Abstract

The application of nanoparticle technology is rapidly expanding. The reduced dimensionality of nanoparticles can give rise to changes in chemical and physical properties, often resulting in altered toxicity. People are exposed dermally to titanium dioxide (TiO_2_) nanoparticles in industrial and residential settings. The general public is increasingly exposed to these nanoparticles as their use in cosmetics, sunscreens and lotions expands. The toxicity of TiO_2_ nanoparticles towards human skin cells is unclear and understudied. We used a human skin fibroblast cell line to investigate the cytotoxicity and clastogenicity of TiO_2_ nanoparticles after 24 h exposure. In a clonogenic survival assay, treatments of 10, 50 and 100 μg/cm_2_ induced 97.8, 88.8 and 84.7% relative survival, respectively. Clastogenicity was assessed using a chromosomal aberration assay in order to determine whether TiO_2_ nanoparticles induced serious forms of DNA damage such as chromatid breaks, isochromatid lesions or chromatid exchanges. Treatments of 0, 10, 50 and 100 μg/cm_2_ induced 3.3, 3.0, 3.0 and 2.7% metaphases with damage, respectively. No isochromatid lesions or chromatid exchanges were detected. These data show that TiO_2_ nanoparticles are not cytotoxic or clastogenic to human skin cells.

## Introduction

Nanoparticles are loosely defined as particles with dimensions less than 100 nm. The small volume and large relative surface area of nanoparticle scan give rise to a number of properties that deviate dramatically from those found in their bulky counterparts [1]. These include changes in thermal behavior, material strength, solubility, conductivity, optical properties and catalytic activity [1–3]. A surge of new products containing nanoparticles has resulted from the application of these properties in commercial, industrial and biomedical products. The Project on Emerging Nanotechnologies reports that as of October 2013, 1,628 consumer products contained nanotechnology [4].

The high photocatalytic and super-hydrophilic properties of titanium dioxide (TiO_2_) nanoparticles have made them popular for a wide variety of applications. The current major applications of TiO_2_ nanoparticles includes; self-cleaning cements, glass and paints; water purification systems; anti-fogging coatings for glass; and as a UV-attenuating ingredient in lotions, sunscreens and cosmetics [5,6]. Robichaud et al. [7] estimate that the increasing demand for TiO_2_ nanoparticles will result in the total conversion of the TiO_2_ industry to nano by 2025, with an annual production of ~ 2.5 million metric tons.

However, the unique properties of TiO_2_ nanoparticles may alter the way they interact with biological molecules and consequently, their toxicity. While bulky TiO_2_ particles have traditionally been considered biologically inert, the toxicity of nano-sized TiO_2_ particles is unclear. The results of studies investigating the genotoxicity of TiO_2_ nanoparticles are contradictory. *In vivo* and *in vitro* studies indicate an increase in oxidative DNA damage, single strand DNA breaks, or micronuclei following exposure to TiO_2_ nanoparticles [8–13]. However, other studies contradict these findings [14–17]. The only clear conclusion from the collective studies on TiO_2_ toxicity is that the crystalline form, particle size and agglomeration of the nanoparticles all play a crucial role in the determining their toxicity.

While these previous studies have been conducted in a variety of cell types, few studies have investigated the effect of TiO_2_ nanoparticles after dermal exposure or in human skin cells. Dermal exposure to TiO_2_ nanoparticles occurs in industrial and residential settings. For example, construction workers and contractors are exposed to TiO_2_ nanoparticles in cement dust, paints and primers. In a residential setting, people are exposed to TiO_2_ nanoparticles through the daily application of lotions, sunscreens and cosmetics. Thus, dermal exposure is a significant route of exposure to the general public as sunscreen alone is utilized by more than 200 million Americans [5].

Studies have shown that TiO_2_ nanoparticles can penetrate the cellular membrane of skin cells [18–21]. Monteiro-Riviere et al. [22] showed that UVB-damage enhanced the penetration of TiO_2_ nanoparticles in sunscreen formulations into the dermal layers. Determining the toxicity of TiO_2_ nanoparticles in human skin cells is crucial to defining whether they represent a human health concern.

The few studies that have investigated the genotoxicity of TiO_2_ nanoparticles in human skin cells offer contradictory results. One study showed an increase in DNA strand breaks and oxidative damage after 6 hours exposure to TiO_2_ nanoparticles, determined by an alkaline Comet assay, with and without Fpg treatment [23]. Other studies found an increase in the activity of key DNA double strand break repair proteins after 24 hours TiO_2_ nanoparticle exposure, measured by immunofluorescence and western blotting for phosphorylated H2A.X, phosphorylated ATM and phosphorylated Chk2 [24,25]. However, a third study showed no alteration of the cell cycle and no increase in apoptosis after 24 hours exposure to TiO_2_ nanoparticles, suggesting that the cell is not halting its cycle to repair DNA damage nor is it inducing death due to such damage [26].

Chromosomal aberrations are a standard component of hazard characterization and are a well-established short term marker for cancer [27]. However, no studies have investigated the ability of TiO_2_ nanoparticles to induce chromosomal aberrations in human skin cells. Thus, the purpose of this study was to investigate the ability of TiO_2_ nanoparticles to induce clastogenicity, expressed as chromosomal aberrations in human skin fibroblasts.

## Materials and methods

### Chemicals and reagents

DMEM and Ham’s F12 50:50 mixture and GlutaGRO (L-alanyl-L-glutamine solution) were purchaed from Mediatech Inc (Herndon, VA). Cosmic Calf Serum was purchased from Hyclone (Logan, UT). All plasticware was purchased from BD Biosciences (Franklin Lakes, NJ). Dulbecco’s phosphate buffered saline (PBS), Gurr’s buffer, penicillin/streptomycin, sodium pyruvate and trypsin/EDTA were purchased from Life Technologies (Grand Island, NY). Crystal violet, calcium chloride, demecolchicine, lead chromate (PbCrO_4_) and potassium chloride were purchased from Sigma-Aldrich (St. Louis, MO). Acetic acid and methanol were purchased from J.T. Baker (Phillipsburg, NJ). Giemsa stain was purchased from Biomedical Specialties Inc. (Santa Monica, CA). Titanium dioxide nanoparticles, Aeroxide TiO_2_ P25, were purchased from Nippon Aerosil Co, LTD (Tokyo, Japan). Microscope slides and cytoseal 60 slide mounting medium were purchased from Thermo Fisher Scientific Inc. (Waltham, MA). Ethyl alcohol, Embed-812 kit, flat embedding mold, gluteraldehyde, osmium tetra oxide, sodium cacodylate, sodium maleate and uranyl acetate were purchased from EMS (Hatfield, PA).

### Cell culture

For these studies we used primary human skin fibroblasts (BJ cells) and human skin fibroblast cells immortalized with hTERT (BJhTERT cells) previously described in Vaziri and Benchimol [28]. Cells were cultured as adherent monolayers of cells in DMEM/F12 50 : 50 mixture, supplemented with 15% cosmic calf serum, 2 mM GlutaGRO, 100 U/ml penicillin/100 μg/ml streptomycin and 0.1 mM sodium pyruvate and subcultured at least once a week. Cells were maintained in a 5% CO_2_ - humidified environment at 37°C.

### Nanoparticle preparation and exposure

The TiO_2_ nanoparticles utilized were Aeroxide TiO_2_ P25, which have been used in many previous studies [10,12,20,25,29,30]. The TiO_2_ nanoparticles were 25 nm in size and spherical in shape with a zeta potential of −36.4 mV as determined by dynamic light scattering (DLS) and transmission electron micrograph (TEM). The crystalline composition of Aeroxide TiO_2_ P25 nanoparticles has been shown to vary between 78–85% anatase, 14–17% rutile and 0–13% amorphous [31] and the surface area has been reported as 46 m^2^/g [10]. The nanoparticles were uncoated and received no physical modification after purchase.

TiO_2_ nanoparticles were suspended in deionized water at a concentration of 50 mg/ml. The nanoparticles were probe sonicated (Misonix Sonicator Ultrasonic Processor XL) at 10 KHz for 5 min directly prior to dilution to ensure an even distribution of the nanoparticles. Dilutions were prepared in cold deionized water filtered with a 0.22 μm filter. Cells were treated with TiO_2_ nanoparticles in low light conditions and incubated in the dark throughout each experiment. Lead chromate (CAS# 7758-97-6, ACS reagent minimum 98% purity), was used as a positive control and administered as a suspension of micro-particles as previously described [32].

The concentrations of TiO_2_ nanoparticles utilized in this study are representative of real-world exposure. The Skin Cancer Foundation reports that most people use one quarter the recommended amount (30 ml) of sunscreen [33]. Most sunscreens contain 2–15% TiO_2_ nanoparticles [5]. If a person applies 7.5 ml of sunscreen containing 2% TiO_2_ nanoparticles (20 mg/ml), they are exposed to 150 mg of nanoparticles. Considering that this volume would be spread over the surface of the skin, each cell would be exposed to a smaller percentage of TiO_2_ nanoparticles. At 1% of the total, each cell would be exposed to 200 μg/ml TiO_2_ nanoparticles, which is the mid-point of our concentration range (0–423 μg/ml). Additionally, Prasad et al. [24] calculated that the average skin exposure to TiO_2_ nanoparticles from sunscreen application would range from 25–75 μg/cm^2^, further validating our range of concentrations (0–100 μg/cm^2^).

### Characterization of TiO_2_ nanoparticles

A transmission electron micrograph (TEM) image was taken of the TiO_2_ nanoparticles in water prior to treatment. To characterize the size of the TiO_2_ nanoparticles under experimental conditions, 90,000 cells were seeded into 2.3 ml cell culture medium per well of 6-well plates (9.5 cm^2^ surface area per well). Cells were allowed to rest for 48 hours to enter log phase growth before treatment with 0, 10, 50 or 100 μg/cm^2^ TiO_2_ nanoparticles. After 24 hours incubation, the extracellular medium was collected. TiO_2_ nanoparticle suspensions were characterized in solution by dynamic light scattering (DLS) using a Malvern NanoZS where particle size distributions were determined on the basis of number, volume and scattering intensity. DLS measurements were also taken for TiO_2_ nanoparticle suspensions in complete media (serum-containing) and media without serum, without cells present.

### Cytotoxicity assay

Cytotoxicity was assessed using a clonogenic survival assay based on our published methods [32]. Briefly, 90,000 cells were seeded into 2.3 ml cell culture medium per well of 6-well plates (9.5 cm^2^ surface area per well). Cells were allowed to enter log phase growth and were treated TiO_2_ nanoparticles or lead chromate (positive control) for 24 hours. Following treatment, cells were harvested, counted and reseeded at a density of 2,000 cells per 100 mm dish (4 dishes per treatment). Cells were allowed to grow to form colonies. Once colonies formed, they were stained with crystal violet and counted. At least 3 independent experiments were conducted.

### Clastogenicity assay

Clastogenicity was assessed by measuring chromosomal aberrations according to our published methods [32]. 500,000 cells were seeded into 13 ml cell culture medium in 100 mm dishes (55 cm^2^ surface area per dish). Cells were allowed to rest for 48 hours to enter log phase growth, followed by treatment with TiO_2_ nanoparticles or lead chromate (positive control) for 24 hours. One hour prior to the end of treatment, demecolcine was added to arrest cells in metaphase. Cells were then harvested, swollen with 0.075 M KCl for 17 min and fixed with 3:1 methanol : acetic acid. Cells were then dropped on clean, wet slides and scored for chromosome abberrations. Three independent experiment were conducted and 100 metaphases were analyzed for each treatment. Chromosome aberrations were scored using a previously defined criteria [32]. DNA damage was expressed as the percent of metaphases with damage and as total aberrations which considers the metaphase and each chromosome as the unit, respectively.

### Uptake of TiO_2_ nanoparticles

The intracellular uptake of TiO_2_ nanoparticles was confirmed with TEM imaging. A monolayer of cells was treated with 50 μg/cm^2^ TiO_2_ nanoparticles for 24 hours. After treatment, cells were harvested, pelleted and resuspended with 5% gluteraldehyde in 0.1M sodium cacodylate buffer for 2 hours. After washing twice with cacodylate buffer, the cell pellet was encapsulated with sodium alginate and calcium chloride, post-fixed with 1% osmium tetraoxide in cacodylate buffer for 2 hours, washed with sodium maleate and stained in 0.5% uranyl acetate for 2 hours in the dark. Cells were then dehydrated with graded ethanol (50% to 100%), followed by propylene oxide. Finally, propylene oxide was gradually replaced with EMbed 812, embedded and cured at 60°C overnight. 30–40 nm sections were stained with uranyl acetate and lead citrate for contrast and examined with Zeis/LEO 922 Omega Transmission Electron Microscope at 100 to 120 kV.

### Statistical analysis

Results were expressed as mean +/− SE of three independent experiments. Standard errors for each mean value were calculated based on the unbiased estimate of variance. Differences among means were evaluated using a Student’s t-test and 95% confidence limits. The criterion for statistical significance was p<0.05. All analyses were conducted using GraphPad QuickCalcs.

## Results

### TiO_2_ nanoparticle aggregation size increases in a concentration-dependent manner

**Figure 1:**
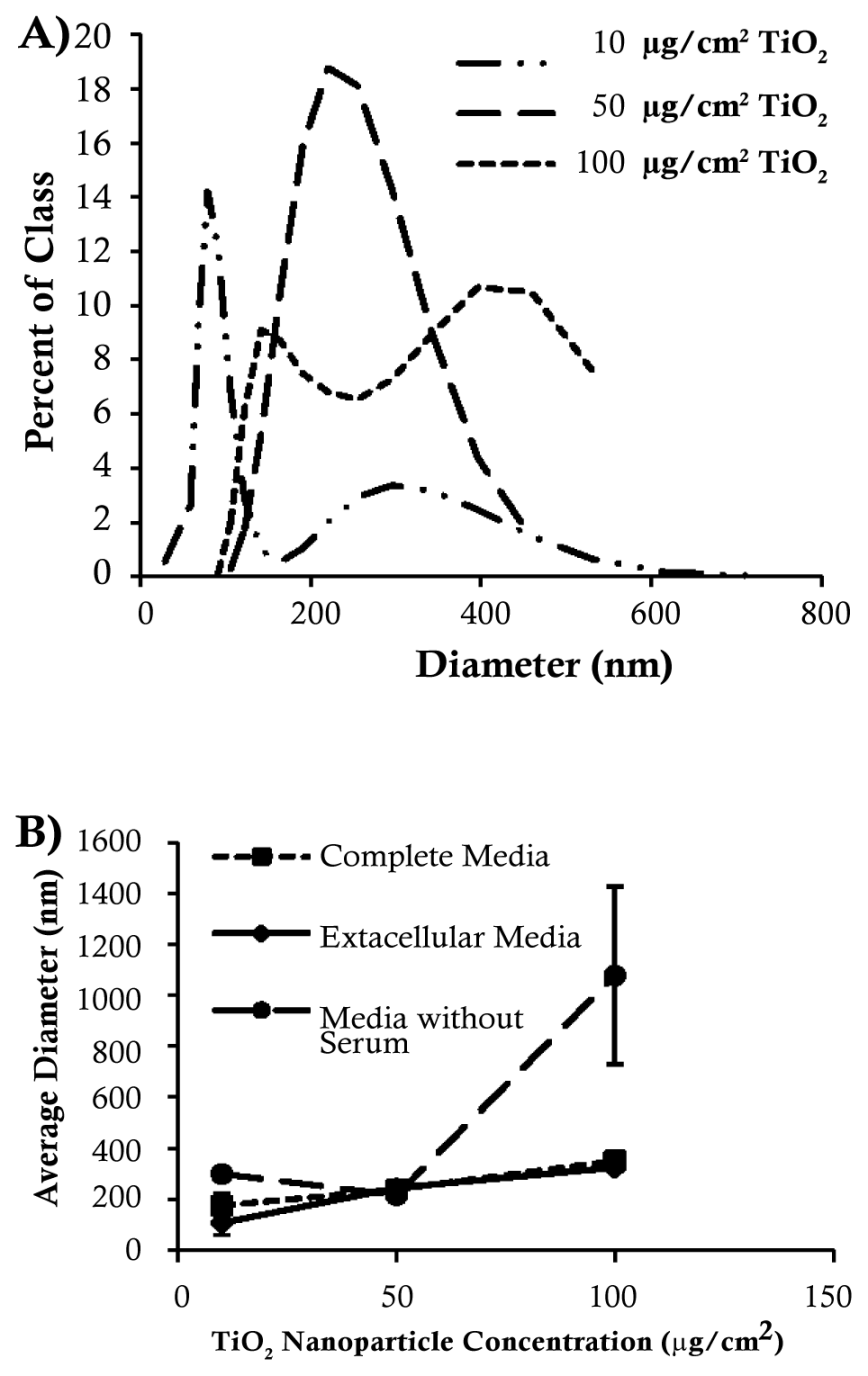
Titanium dioxide nanoparticle size distributions based on DLS This figure shows that the average size distribution of agglomerated TiO_2_ nanoparticles increases in a concentration dependent manner. Data represent the mean of 3 independent experiments +/− standard error of the mean. A. Comparison of the size distributions of TiO_2_ nanoparticles in extracellular media. B. Comparison of the average diameter of TiO_2_ nanoparticles aggregates in extracellular media, complete media and media without serum.

To determine the size of the agglomerated TiO_2_ nanoparticles that the cells would be exposed to, the size distribution of the nanoparticle agglomerations was measured in the extracellular medium of cells treated with 10, 50 or 100 μg/cm^2^ TiO_2_ nanoparticles using dynamic light scattering (DLS) and analyzed based on the number distribution. The peak distribution of TiO_2_ nanoparticle agglomeration was less than 100 nm when cells were treated with 10 μg/cm^2^ TiO_2_ nanoparticles. However, the distribution showed a peak at 225 nm when cells were treated with 50 μg/cm^2^ TiO_2_ nanoparticles and there was a broad size distribution from 150 nm to 500 nm when cells were treated with 100 μg/cm^2^ TiO_2_ nanoparticles (Figure 1A). The TiO_2_ aggregates were comprable in size in the complete medium and the extracellular medium while they were larger in the serum-free media (Figure 1B).

### TiO_2_ nanoparticles are not cytotoxic to human skin fibroblasts

**Figure 2:**
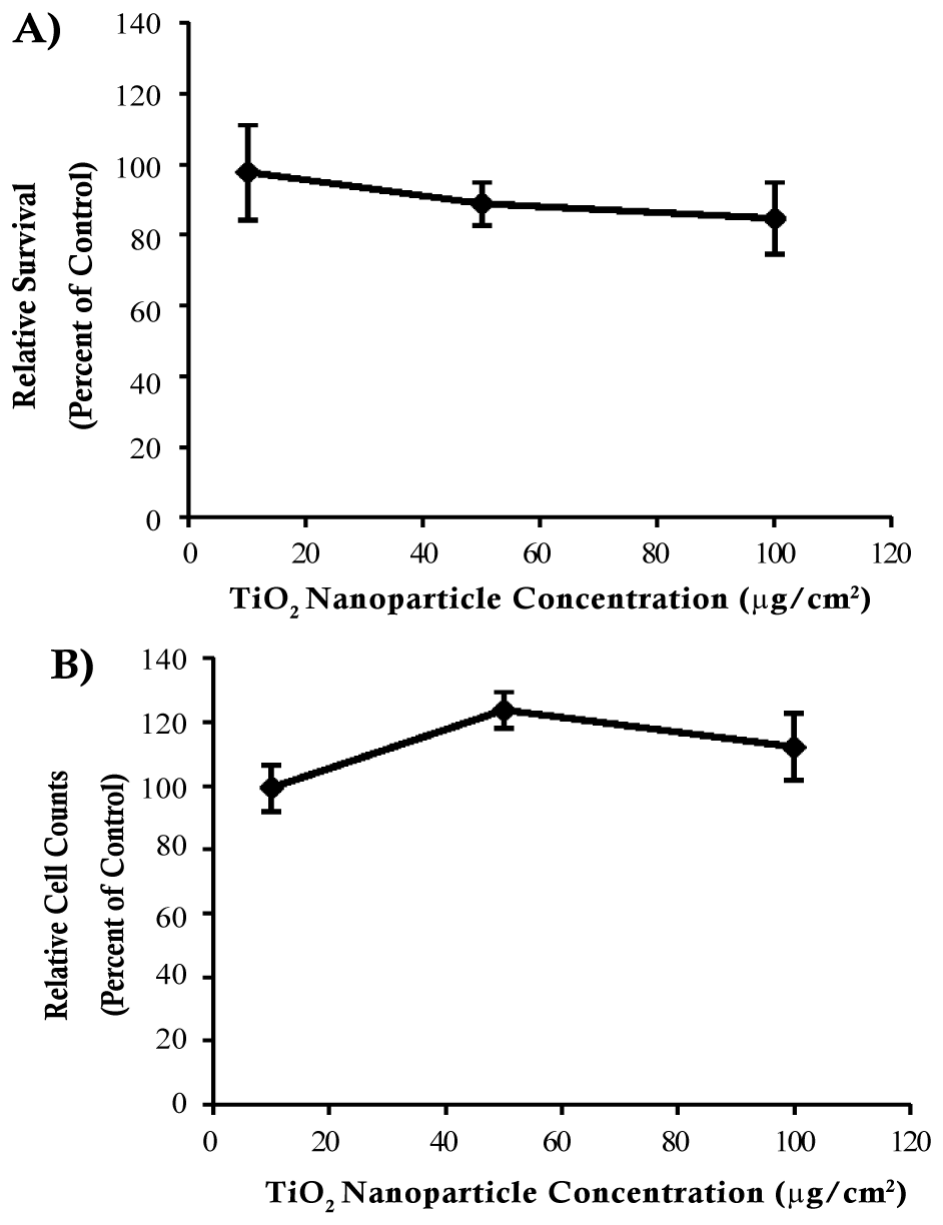
TiO_2_ nanoparticles were not cytotoxic to human skin fibroblasts This figure shows that TiO_2_ nanoparticles did not induce cytotoxicity in human skin fibroblasts measured by a clonogenic survival assay. A. cell count. B. Data represent the mean of 3 independent experiments +/− standard error of the mean.

TiO_2_ nanoparticles did not induce cytotoxicity in human skin fibroblasts. In the clonogenic survival assay, treatments of 10, 50 and 100 μg/cm^2^ induced 97.8, 88.8 and 84.7% relative survival (percent of control), respectively (Figure 2A). There was no significant difference between the relative survival of the treated cells and the control cells. Cell survival was also evidenced by cell counts after 24 hours TiO_2_ treatment. Treatments of 10, 50 and 100 μg/cm^2^ induced 99.4, 123.8 and 112.1% relative cell counts (percent of control), respectively (Figure 2B). These results were confirmed in primary human skin fibroblasts. In contrast, 0.5 μg/cm^2^ lead chromate (positive control) induced 0.9% relative survival in the clonogenic assay and 80.3% relative cell count, indicating that the assays were functional (data not shown).

### TiO_2_ nanoparticles are not clastogenic to human skin fibroblasts

**Figure 3:**
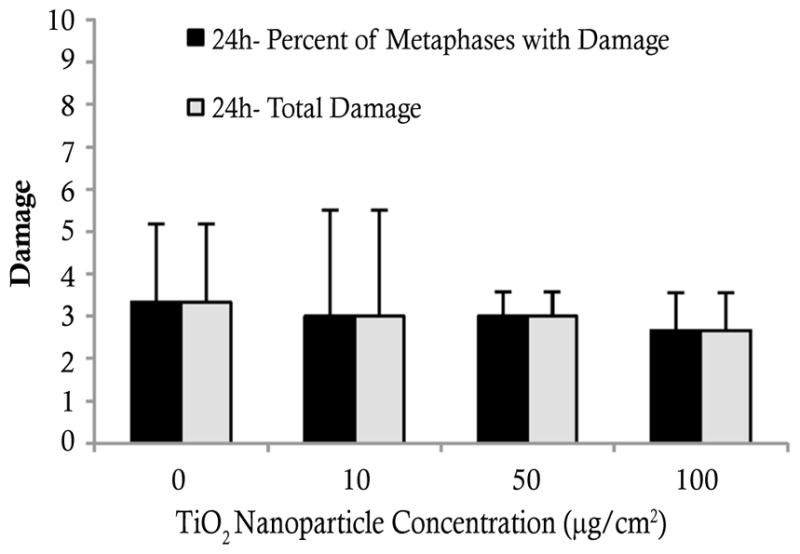
TiO_2_ nanoparticles were not clastogenic to human skin fibroblasts This figure shows that TiO_2_ nanoparticles did not induce chromosomal aberrations in human skin fibroblasts. Data represent the mean of 3 independent experiments +/− standard error of the mean.

TiO_2_ nanoparticles did not induce clastogenicity, measured as chromosomal aberrations, in human skin fibroblasts (Figure 3). Treatments of 0, 10, 50 and 100 μg/cm^2^ induced 3.3, 3.0, 3.0 and 2.7% metaphases with damage, respectively. The same amount of damage was observed when represented as total abberrations in 100 metaphases. There was no significant difference between the chromosomal damage of the treated cells and the control cells. No isochromatid lesions or chromatid exchanges were identified during the chromosomal analysis. These results were confirmed in primary human skin fibroblasts. The positive control, 0.5 μg/cm^2^ lead chromate, indicated that our assay was functional as it induced 29.7% metaphases with damage and 44 total aberrations (data not shown).

### TiO_2_ nanoparticles are internalized by human skin fibroblasts

**Figure 4:**
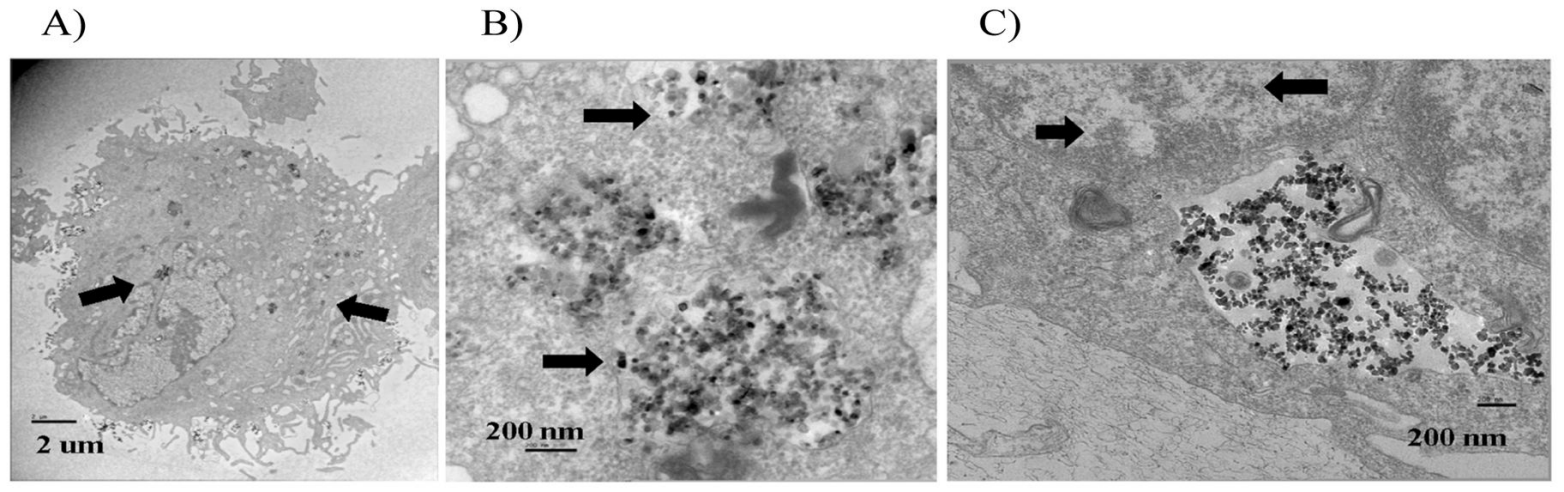
TiO_2_ nanoparticles were internalized by human skin fibroblasts This figure shows that TiO_2_ nanoparticles were internalized by human skin fibroblasts shown by TEM imaging. The black arrows indicate TiO_2_ nanoparticle aggregates within different subcellular compartments. A. TEM image showing TiO_2_ nanoparticles located throughout the entire cell (4,000X). B. TEM image showing different sizes of TiO_2_ nanoparticle aggregates within the cytoplasm (10,000X). C. TEM image showing TiO_2_ nanoparticles within the nucleus (10,000X).

TEM imaFging showed that TiO_2_ nanoparticles were internalized by human skin fibroblasts (Figure 4A). TiO_2_ nanoparticles were identified in the cytoplasm, often associated with lysosomes (Figure 4B), and in the nucleus (Figure 4C).

## Discussion

TiO_2_ nanoparticles are widely utilized for their unique super-hydrophilic and photocatalytic properties. In addition to industrial exposure through cement dusts, paints and primers, the general public is exposed to TiO_2_ nanoparticles through its inclusion in various personal products. TiO_2_ nanoparticles are utilized in sunscreens, cosmetics and lotions resulting in daily dermal exposure. For example, it is estimated that 33 million Americans use sunscreen daily and another 177 million people use it occasionally [5]. However, the toxicity of TiO_2_ nanoparticles in skin cells is understudied and not well defined.

To our knowledge, this is the first study to measure the ability of TiO_2_ nanoparticles to induce chromosome aberrations in a human cell line. Our data show no significant increase in aberrations after 24 hours exposure. This result is consistent with previously published studies that found TiO_2_ nanoparticles did not induce chromosome aberrations in CHO cells [15,17]. Previous studies that have measured genotoxicity with other assays further support this finding. TiO_2_ nanoparticles did not induce a significant increase in DNA strand breaks in human lung epithelial cells, lung fibroblasts or lymphoblasts, measured by Comet assay [11,14,16,29].

DNA double strand breaks are a serious form of DNA damage that can lead to chromosomal aberrations if they are not repaired before the cell enters mitosis. Jugan et al. [10] showed that TiO_2_ nanoparticles do not induce DNA double strand breaks, measured as phosphorylated H2A.X. In agreement with this, another study indicated no response of the DNA double strand break repair proteins, ATM and RAD51after TiO_2_ exposure [16]. In contrast, however, other studies show a concentration-dependent increase in DNA double strand breaks, measured as phosphorylated H2A.X and the activation of the DNA double strand break repair proteins, ATM and Chk2 [24,25]. It is important to note that the phosphorylation of H2A.X is an indirect measure of DNA double strand breaks. From one perspective, phosphorylated H2A.X represents an early event in the DNA double strand break repair signaling pathway [34]. Therefore, an increase in the phosphorylation of H2A.X indicates the activation of this repair pathway. If TiO_2_ nanoparticles induce DNA double strand breaks, the activation of DNA double strand break repair would prevent this damage from causing genomic alterations or chromosomal aberrations. Our data suggest that, if this repair perspective is correct, any DNA breaks formed after 24 hours TiO_2_ nanoparticle exposure are successfully repaired before they manifest as chromosomal aberrations.

An alternative perspective for phosphorylated H2A.X is that it also serves as a marker of senescence [35]. Thus, rather than measuring breaks, the H2A.X foci or some fraction of them, may actually reflect senescent cells. This possibility is consistent with the observed differences in cytotoxicity between the two studies measuring H2A.X, with one measuring increased phosphorylated H2A.X thus finding increased senescence and consequently an increase in their apparent cytotoxicity measures and the other finding no increase in H2A.X and no toxicity [10,24]. If this possibility were correct, it would be consistent with our observations of no chromosome damage in skin cells and others observations of no DNA damage when using direct measures of damage in other cell types [11,14,15,16,17,29].

Our data showed that TiO_2_ nanoparticles did not induce cytotoxicity after 24 hours exposure. This outcome is consistent for TiO_2_ nanoparticles when determined by a clonogenic survival assay, as we used in our study [10,29]. Our results are also in agreement with other reports that found no significant decrease in cell survival after 24 hours exposure in human lung epithelial cells (A549 and BEAS-2B) and lung fibroblasts, measured with SRB, Trypan blue or the MTT assay [9,14,16].

In contrast, several studies have shown that TiO_2_ nanoparticles induce cell death measured by Trypan blue or the MTT assay in human skin fibroblasts, epidermal (A431) and bronchial epithelial cells (BEAS-2B) [8,23,24]. Magdolenova et al. [29] suggested that the method of dispersing the nanoparticles prior to treatment may affect the resulting toxicity. In their study, two different methods of dispersal were utilized that resulted in (1) a stable dispersion of TiO_2_ nanoparticles and (2) an unstable dispersion of TiO_2_ nanoparticles with a larger agglomerate size. The unstable dispersion induced cytotoxicity in monkey kidney fibroblasts (Cos-1), while the stable dispersion did not.

Interestingly, one previous study has utilized two different cytotoxicity assays in the same cell line after exposure to the same TiO_2_ nanoparticles and obtained different results [10]. The study measured cytotoxicity in lung epithelial cells (A549) after exposure to TiO_2_ nanoparticles varying in range from 12 – 140 nm. After 48 hours exposure, they reported a significant decrease in cell viability measured by the MTT assay, but no significant decrease measured by a clonogenic proliferation assay. Although the results of the MTT assay were significant, the authors note that even the most cytotoxic TiO_2_ nanoparticles induced less than 25% cell death [10]. It is interesting that the significant result of the MTT assay did not represent a large decrease in cell viability.

The clonogenic survival assay is a gold standard among cytotoxicity assays. As it depends on the survival and proliferation of cells, it is more sensitive than the other cytotoxicity assays mentioned above [36]. Therefore, the clonogenic survival assay should be more frequently included in the hazard characterization of TiO_2_.

One might consider that the differences between our results and the previous study conducted in skin cells are due to a difference in cell line. Our skin fibroblasts were immortalized with hTERT, while the previous study used primary skin fibroblasts [24]. It seems unlikely that the hTERT would have much of an effect on the results as previous data show hTERT immortalization does not affect the cellular response to particulate metal exposure [37]. In addition, we did confirm our results in primary skin fibroblasts and found the same outcome (data not shown).

In our study, the TiO_2_ nanoparticles agglomerated to form aggregates up to twenty times larger than the original nanoparticle size of 25 nm. The concentration-dependent increase in TiO_2_ nanoparticle aggregation was observed in all sample types and therefore cannot be due to a cellular mechanism. The TiO_2_ aggregates were nearly identical in size in the complete medium and the extracellular medium while they were larger in the serum-free media. Previous studies have shown that the presence of serum inhibits the aggregation of TiO_2_ and other nanoparticles [30,38]. These results indicate that even though the TiO_2_ nanoparticles were sonicated directly before treatment in order to evenly disperse the nanoparticles, the cells are subjected to TiO_2_ nanoparticle agglomerations of increasing size in a concentration-dependent manner.

It has previously been recognized that data characterizing nanoparticles is crucial in order to compare results of different studies [39,40]. However, most studies only characterize nanoparticles at one concentration. Our findings suggest that nanoparticle characterization at the lowest and highest concentrations would enhance our interpretation of toxicity data by providing the range of agglomeration present.

Our data showed that TiO_2_ nanoparticles can penetrate into in the cytoplasm and nucleus of human skin fibroblast cells after 24 hours exposure. These results are in agreement with previous *in vitro* studies [18,19]. Considered together, the results of this study indicate that TiO_2_ nanoparticles do not induce cytotoxicity or chromosomal aberrations after 24 hours exposure, even though they penetrate both the cellular and nuclear membranes.

Real-world dermal exposure to TiO_2_ nanoparticles most likely occurs for periods of time less than that utilized in this study (24 hours). Workers exposed to TiO_2_ nanoparticles in cement dust and paints probably wash this from their skin within 24 hours. Likewise, sunscreens, lotions and cosmetics are usually washed from the skin with a day. However, dermal exposure to TiO_2_ nanoparticles is repetitive with many people enduring subsequent exposures on a daily basis. Future studies are needed to determine the effects of repeated, short-term exposure to TiO_2_ nanoparticles.

## Abbreviations

TiO_2_: Titanium dioxide
